# Subcutaneous versus transvenous implantable cardioverter-defibrillator among drug-induced type-1 ECG pattern Brugada syndrome: *a propensity score matching analysis from IBRYD study*

**DOI:** 10.1007/s00380-022-02204-x

**Published:** 2022-11-24

**Authors:** Vincenzo Russo, Alfredo Caturano, Federico Guerra, Federico Migliore, Giuseppe Mascia, Andrea Rossi, Martina Nesti, Vincenzo Ezio Santobuono, Emilio Attena, Gianfranco Tola, Luigi Sciarra, Giulio Conte, Alessandro Paoletti Perini, Pietro Francia, Gregory Dendramis, Zefferino Palamà, Stefano Albani, Andrea Ottonelli Ghidini, Leonardo Calò, Antonio D’Onofrio, Enrico Baldi, IBRYD Study Group, Gerardo Nigro, Ferdinando Carlo Sasso, Luca Barca, Italo Porto, Pasquale Notarstefano, Maria Antonietta Ruocco, Livia Franchetti Pardo, Carmen Adducci, Nicola Berlier, Berardo Sarubbi, Alessandro Vicentini, Roberto Floris, Emanuele Romeo, Paolo Golino

**Affiliations:** 1 Department of Medical Translational Sciences, Division of Cardiology, University of Campania “Luigi Vanvitelli”, Monaldi Hospital, Naples, Italy; 2 Department of Advanced Medical and Surgical Sciences, University of Campania “Luigi Vanvitelli”, Piazza Luigi Miraglia 2, T80138 Naples, Italy; 3 Azienda Ospedaliera Universitaria Ospedali Riuniti, Ancona, Italy; 4 Università degli Studi di Padova, Padua, Italy; 5 IRCCS San Martino Polyclinic Hospital, Genoa, Italy; 6 Gabriele Monasterio Foundation, Pisa, Italy; 7 Cardiovascular and Neurological Department, Ospedale San Donato, Via Nenni, 20/22, 52100 Arezzo, Italy; 8 Department of Interdisciplinary Medicine and Policlinico of Bari, Cardiology Unit, University of Bari “Aldo Moro”, Bari, Italy; 9 Cardiology Unit, Roccadaspide Hospital, ASL Salerno, Roccadaspide, Italy; 10 Azienda Ospedaliera Brotzu, Cagliari, Italy; 11 Policlinico Casilino, Cardiology Unit, Rome, Italy; 12 Cardiocentro Ticino Foundation, Lugano, Switzerland; 13 Azienda Sanitaria di Firenze, Florence, Italy; 14 Azienda Ospedaliero-Universitaria Sant’Andrea, Rome, Italy; 15 Clinical and Interventional Arrhythmology, Cardiology Unit, ARNAS, Ospedale Civico Di Cristina Benfratelli, Palermo, Italy; 16 Casa di Cura Villa Verde, Taranto, Italy; 17 Umberto Parini Regional Hospital, Aosta, Italy; 18 Versilia Hospital, Cardiology Unit, Lido di Camaiore (LU), Italy; 19 Departmental Unit of Electrophysiology, Evaluation and Treatment of Arrhythmias, Monaldi Hospital, Naples, Italy; 20 IRCCS Policlinico San Matteo, Pavia, Italy; 21 Clinical Cardiology, Department of Medical Sciences and Public Health, University of Cagliari, Cagliari, Italy; 22 Department of Cardiology, University of Campania “Luigi Vanvitelli”, Monaldi Hospital, Naples, Italy

**Keywords:** Brugada syndrome, Drug-induced type 1 Brugada syndrome, Implantable cardioverter-defibrillator, Sudden cardiac death, Subcutaneous cardioverter-defibrillator, Transvenous cardioverter-defibrillator, Inappropriate shock, ICD-related infection, ICD-related complication

## Abstract

No real-world data are available about the complications rate in drug-induced type 1 Brugada Syndrome (BrS) patients with an implantable cardioverter-defibrillator (ICD). Aim of our study is to compare the device-related complications, infections, and inappropriate therapies among drug-induced type 1 BrS patients with transvenous- ICD (TV-ICD) versus subcutaneous-ICD (S-ICD). Data for this study were sourced from the IBRYD (Italian BRugada sYnDrome) registry which includes 619 drug-induced type-1 BrS patients followed at 20 Italian tertiary referral hospitals. For the present analysis, we selected 258 consecutive BrS patients implanted with ICD. 198 patients (76.7%) received a TV-ICD, while 60 a S-ICD (23.4%). And were followed-up for a median time of 84.3 [46.5–147] months. ICD inappropriate therapies were experienced by 16 patients (6.2%). 14 patients (7.1%) in the TVICD group and 2 patients (3.3%) in S-ICD group (*log-rank P* = *0.64*). ICD-related complications occurred in 31 patients (12%); 29 (14.6%) in TV-ICD group and 2 (3.3%) in S-ICD group (*log-rank P* = *0.41*). ICD-related infections occurred in 10 patients (3.88%); 9 (4.5%) in TV-ICD group and 1 (1.8%) in S-ICD group (*log-rank P* = *0.80*). After balancing for potential confounders using the propensity score matching technique, no differences were found in terms of clinical outcomes between the two groups. In a real-world setting of drug-induced type-1 BrS patients with ICD, no significant differences in inappropriate ICD therapies, device-related complications, and infections were shown among S-ICD vs TV-ICD. However, a reduction in lead-related complications was observed in the S-ICD group. In conclusion, our evidence suggests that S-ICD is at least non-inferior to TV-ICD in this population and may also reduce the risk of lead-related complications which can expose the patients to the necessity of lead extractions.

## Introduction

The sudden cardiac death (SCD) risk stratification of Brugada syndrome (BrS) patients is still challenging, in particular among those with a drug-induced type I electrocardiographic (ECG) pattern [1, 2]**;** in this subgroup, the implantable cardioverter-defibrillator (ICD) implantation for primary SCD prevention may be considered only in case of inducible ventricular fibrillation (VF) during programmed electrical stimulation at the electrophysiological study (EPS) [3]**.** The transvenous ICD (TV-ICD) implantation was frequently associated with complications among BrS patients, including inappropriate shocks (IAS), device or lead malfunctions, and infections[4–6]**.** Moreover, IAS was frequent in BrS patients with a subcutaneous ICD (S-ICD)[7]**.** However, these evidences refer to study cohort including more likely BrS patients with spontaneous ECG pattern and no data are available about the complications rate among drug-induced BrS patients implanted with TV-ICD or S-ICD. This study aimed to compare the device-related complications and inappropriate shocks among drug-induced BrS patients with TV-ICD versus S-ICD.

## Materials and methods

### Database

Data for this study were sourced from the IBRYD (Italian BRugada sYnDrome) registry which includes 619 drug-induced type-1 BrS patients followed at 20 tertiary referral hospitals throughout the Italian territory. All patients were diagnosed from July 1997 to May 2021, while follow-up was censored in December 2021. For the present analysis, we selected 262 consecutive BrS patients implanted with both subcutaneous (S-ICD Group) and transvenous (TV-ICD Group). From 2015, all participating hospitals have started to routinely implant S-ICD for primary prevention of SCD in young BrS patients not in need of pacing. Patients with incomplete baseline (n: 3) or follow-up data (n: 1) were excluded. The local institutional review boards approved the study (ID 553–19), and all patients provided written informed consent for data storage and analysis.

### Diagnosis and EPS study protocol

BrS diagnosis was performed by inducing a type 1 ECG pattern through a flecainide or ajmaline drug test in all patients. Flecainide (2 mg/kg) was administered over 10 min, while Ajmalina (1 mg/kg) was administered over 5 min. The test was considered positive only if a coved type I ECG was documented in at least 1 of the right precordial leads (V1, V2), placed at the second, third, or fourth intercostal space. The EPS protocol consisted of double stimulation, at the apex and at the right ventricular outflow tract, of 2 drives (600 and 400 ms) and up to 3 extrastimuli by decreasing the coupling interval until inducing sustained ventricular arrhythmias, reaching chamber refractoriness, or a minimal coupling interval of 200 ms. The stimulation protocol was discontinued if VF or sustained (30 s)/syncopal polymorphic VT were induced.

### ICD programming

The programming of the parameters for the detection of VT/VF was done according to the guidelines’ recommendations at the time of implant and optimized during the follow-up.

Until 2007, TV-ICDs were programmed with a VT window (cut-off rate 180–220 bpm) and/or a single VF zone (cut-off rate ≥ 220 bpm). From 2007, a single VF detection zone (cut-off rate 222–250 bpm) and a maximum of 6 shocks were programmed. S-ICD devices were programmed with a conditional zone, between 200 and 250 bpm, and a shock zone > 250 bpm.

### Endpoints

The primary study endpoints were: ICD inappropriate therapies, defined as anti-tachycardia pacing (ATP) and/or shocks for conditions other than ventricular tachycardia (VT) or ventricular fibrillation (VF); ICD-related complications, defined as all pulse generator (PG) or lead-related complications requiring surgical intervention; ICD-related infections, defined as all systemic infections requiring complete removal of thesystem including the leads extraction. Moreover, the type and distribution of ICD-related complications, defined as early, if occurred within 30 days after ICD implantation, or late, if occurred later than 30 days after implantation, were assessed. The secondary endpoint was all-cause mortality and appropriate ICD therapies.

### Statistical analysis

Categorical data were expressed as number and percentage, whereas continuous variables were expressed as either median [interquartile range (IQR)] or mean ± SD, based on their distribution as assessed both by the Kolmogorov–Smirnov and the Shapiro–Wilk tests. Between-group differences, for categorical variables, were assessed by the chi-square test, with the application of Yates correction where appropriate. Either parametric Student’s t-test or nonparametric Mann–Whitney U test and Wilcoxon test were instead used to compare continuous variables, according to their distribution. The nearest neighbor propensity score matching (PSM) method, with a 1:1 ratio, without replacement, and with the use of a caliper (0.25-SD distance tolerance), was used to minimize the differences in baseline characteristics between patients receiving S-ICD vs TV-ICD group. Covariates included in the model were those significantly different between the 2 groups (age, history of syncope, and alcohol abuse).

Kaplan–Meier analysis was performed to assess the risk of primary outcome events either in the whole population (unadjusted) or in the matched cohorts (adjusted). A time-dependent Cox univariable (unadjusted) and multivariable (adjusted) regression model was used to evaluate the association between S-ICD and clinical outcome events. The multivariable model was computed on all covariates with a p-value < 0.05. A 2-sided probability *p*-value < 0.05 was considered statistically significant. All analyses were performed using SPSS statistical software (version 24.0, SPSS, Chicago, Illinois) and STATA 14.0 software (StataCorp, College Station, Texas).

## Results

### Study population

258 drug-induced BrS patients (mean age 51 ± 15; male 76.4%) with both TV-ICD (*n*: 198, 76.7%) and S-ICD (*n*: 60, 23.4%) followed for a median follow-up of 84.3 [46.5–147] months were included in the study. The indication for ICD implantation was primary prevention in 253 patients (98.1%) and secondary prevention in 5 patients (1.9%). TV-ICD was single or dual chambers in 187 (72.5%) and 10 patients (17.5%), respectively. S-ICD group showed more likely younger age (44 ± 12 vs 53 ± 15 years; *P* < *0.0001*), lower prevalence of history of both syncope (40 *vs* 58.5%; *P* = *0.012*), documented atrial tachyarrhythmias (1.7 *vs* 9.6%; *P* = *0.045*) and alcohol abuse (0 *vs* 6.5%; *P* = *0.04*) compared to TV-ICD group; moreover S-ICD group was followed for less time (46.3 [15.5–71] *vs* 111 [60.6–160]; *P* < *0.0001*). Among TV-ICD patients, 61 patients (mean age 56.4 ± 14.2; male 72.1%) were implanted before 2007 and 131 (mean age 51.9 ± 14.99; male 77.4%) after 2007; no significant difference in baseline clinical characteristics was shown between the two groups. After PSM, 120 patients with balanced baseline characteristics were identified; all patients were implanted with single-lead ICD for primary prevention. All baseline clinical characteristics of the study population are summarized in Table 1.

**Table 1:** Baseline characteristics of the study population

	S-ICD group *n*=60	TV-ICD group *n* = *198*	*P*	Matched TV-ICD group *n* = *60*	*P*
Male gender, *n* (%)	47 (78.3)	150 (75.7)	*0.68*	49 (81.7)	*0.52*
Age (years), mean ± SD	44 ± 12	53 ± 15	< *0.0001*	46 ± 11	*0.17*
Family history of sudden cardiac death, *n* (%)	31 (52)	84 (42.4)	*0.19*	30 (50)	*0.93*
History of Syncope, *n* (%)	24 (40)	116 (58.5)	*0.012*	24 (40)	*0.94*
History of atrial tachyarrhythmias, *n* (%)	1 (1.7)	19 (9.6)	*0.045*	4 (6.7)	*0.86*
Smoking history, *n* (%)	15 (25)	52 (26)	*0.88*	17 (28.3)	*0.64*
Alcohol abuse, *n* (%)	0 (0)	13 (6.5)	*0.04*	3 (5)	*0.11*
Hypertension, *n* (%)	12 (20)	65 (27.7)	*0.22*	16 (26.7)	*0.30*
Dyslipidemia, *n* (%)	13 (21)	54 (27.3)	*0.33*	11 (18.3)	*0.68*
Coronary artery disease, *n* (%)	0 (0)	6 (3)	*0.17*	1 (1.7)	*0.31*
History of cardiac arrest, *n* (%)	0 (0)	5 (2.5)	*0.22*	0 (0)	*0.99*
Sinus Rhythm, *n* (%)	59 (98.3)	181 (91.4)	*0.07*	57 (95)	*0.55*
Left ventricular ejection fraction, mean ± SD	61.2 ± 4	60 ± 4	*0.08*	61 ± 3	*0.38*
Electrophysiological study positivity, *n* (%)	24 (40)	101 (51)	*0.14*	35 (58.3)	*0.08*

n= number, SD= standard deviation, S-ICD= subcutaneous implanted cardiac defibrillator, TV-ICD= transvenous implanted cardiac defibrillator

### Clinical outcome

The primary outcome events, divided into S-ICD vs TV-ICD subgroup are reported in Table 2.

**Table 2:** Primary outcome events at follow-upamongoverall population

	S-ICD group *n* = *60*	TV-ICD group *n* = *198*	*p*
Inappropriate ICD therapies, *n* (%)	2 (3.3)	14 (7.1)	*0.06*
Causes of inappropriate therapies			
T wave oversensing, *n* (%)	0 (0)	1 (0.5)	*0.58*
Myopotential oversensing, *n* (%)	0 (0)	0 (0)	
Atrial Fibrillation, *n* (%)	0 (0)	7 (3.5)	*0.14*
Atrial Tachycardia, *n* (%)	0 (0)	3 (1.5)	*0.34*
Air Entrapments, *n* (%)	2 (3.3)	0 (0)	*0.01*
Noise oversensing, *n* (%)	0 (0)	3 (1.5)	*0.34*
ICD-related complications, *n* (%)	2 (3.3)	29 (14.6)	*0.02*
PG Malfunction, *n* (%)	1 (1.7)	7 (3.5)	*0.46*
Lead related complications, *n* (%)	1 (1.7)	22 (11.1)	*0.02*
Lead failure, *n* (%)	0 (0)	9 (4.5)	*0.09*
Lead dislodgement, *n* (%)	1 (1.7)	4 (2.02)	*0.86*
Lead Fracture, *n* (%)	0 (0)	9 (4.5)	*0.09*
ICD infectious complications, *n* (%)	1 (1.7)	9 (4.5)	*0.31*
Timing of overall patients’ complications			
Early complications, *n* (%)	2 (3.3)	0 (0)	*0.01*
Late complications, *n* (%)	5 (8.3)	44 (22.2)	*0.02*

#### Inappropriate ICD therapies

In the entire population, ICD inappropriate therapies were experienced by 16 patients (6.2%). 14 patients (7.1%) in the TV-ICD group and 2 patients (3.3%) in S-ICD group (*P* = *0.06*). In all cases, the inappropriate ICD therapies were ICD shocks. The annual incident rate of ICD inappropriate therapies was 0.6%. The Kaplan–Meier analysis did not show a significantly different risk of inappropriate ICD therapies between the TV-ICD group implanted before and after 2007 (*log-rank P* = *0.60*). At Cox univariable analysis no baseline patients’ characteristic, including the S-ICD (OR: 0.99; 95% CI 0.22- 4.06; *P* = *0.99*) and the ICD implantation before 2007 (OR: 0.75; 95% CI 0.26- 2.2; *P* = *0.60*) was associated with inappropriate ICD therapies.

**Fig. 1:**
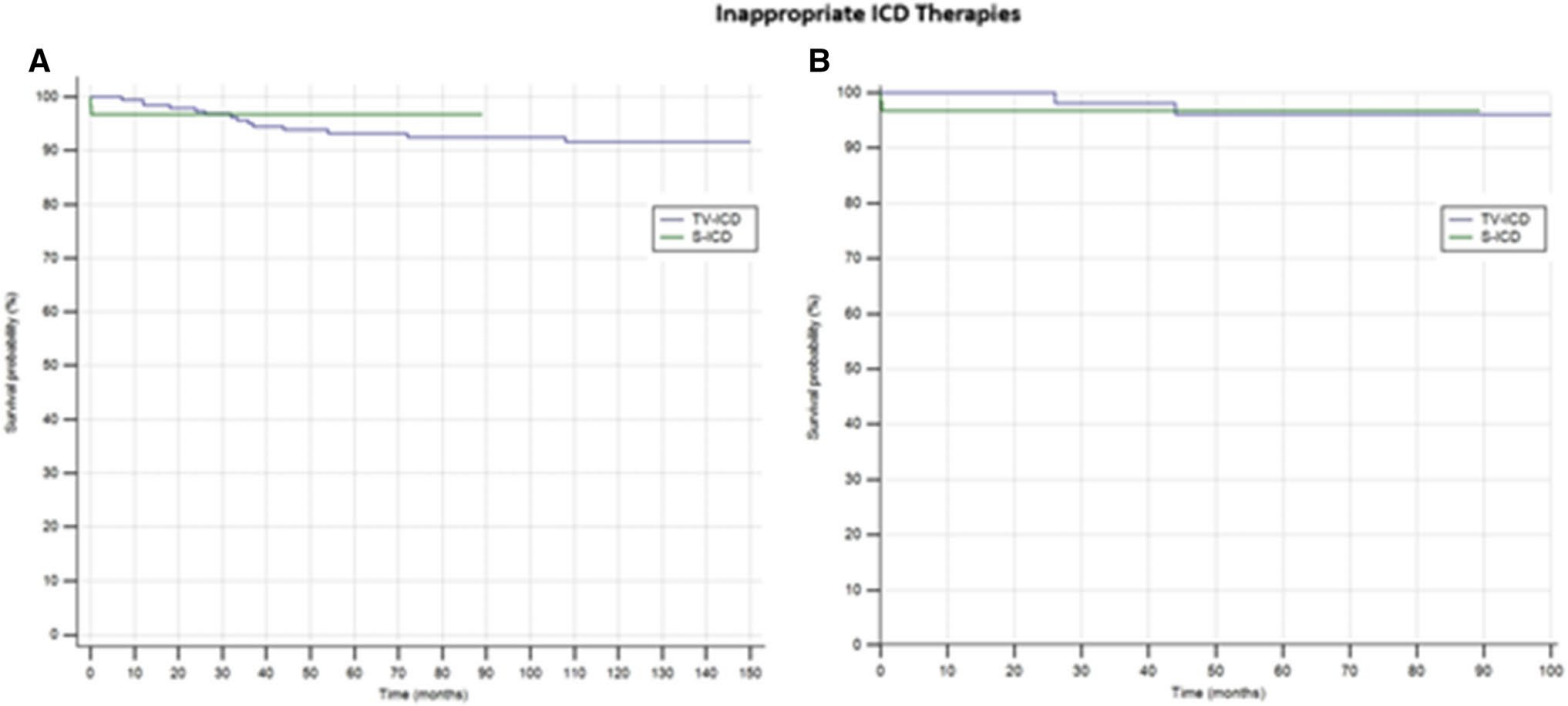
Kaplan–Meier curvecomparing survival from inappropriate ICD therapies among S-ICD vs TV-ICD groups in unmatched (**A**) and matched (**B**) cohorts

In the matched cohort, ICD inappropriate therapies were experienced by 3 patients (2.5%). 2 patients (3.3%) in the S-ICD group, and 2 patient (3.3%) in the TV-ICD group (*P* = *0.986*). At Cox univariable analysis no baseline patients’ characteristic, including the S-ICD (OR: 1.19; 95% CI 0.17–8.6; *P* = *0.86*), was associated with inappropriate ICD therapies.The Kaplan–Meier analysis did not show a significantly different risk of inappropriate ICD therapies between the two subgroups in both unmatched (*log-rank P* = *0.64*) and matched (*log-rank P* = *0.86*) cohorts (Fig. 1).

#### ICD-related complications

ICD-related complications in need of surgical revision occurred in 31 patients (12%); 29 (14.6%) in TV-ICD group and 2 (3.3%) in S-ICD group (*P* = *0.018*); mainly due to increased lead-related complications in TV-ICD vs S-ICD group (14.6% *vs*3.3%; *P* = *0.025).* In contrast, no significant differences were shown in PG-related complications between the two subgroups (3.5% vs 1.7%; *P* = *0.46).* The annual incident rate of ICD-related complications was 1.6%. At Cox univariate analysis no baseline patients’ characteristics, including the S-ICD (OR: 0.53; 95% CI: 0.12- 2.2; *P* = *0.39*), was associated with ICD-related complications.

In the matched cohort, ICD-related complications in need of surgical revision occurred in 13 patients (10.8%); 11 (18.3%) in TV-ICD group and 2 (3.33%) in S-ICD group (*P* = *0.008);* mainly due to increased lead-related complications in TV-ICD vs S-ICD group (15% *vs*1.7%; *P* = *0.008).*

**Fig. 2:**
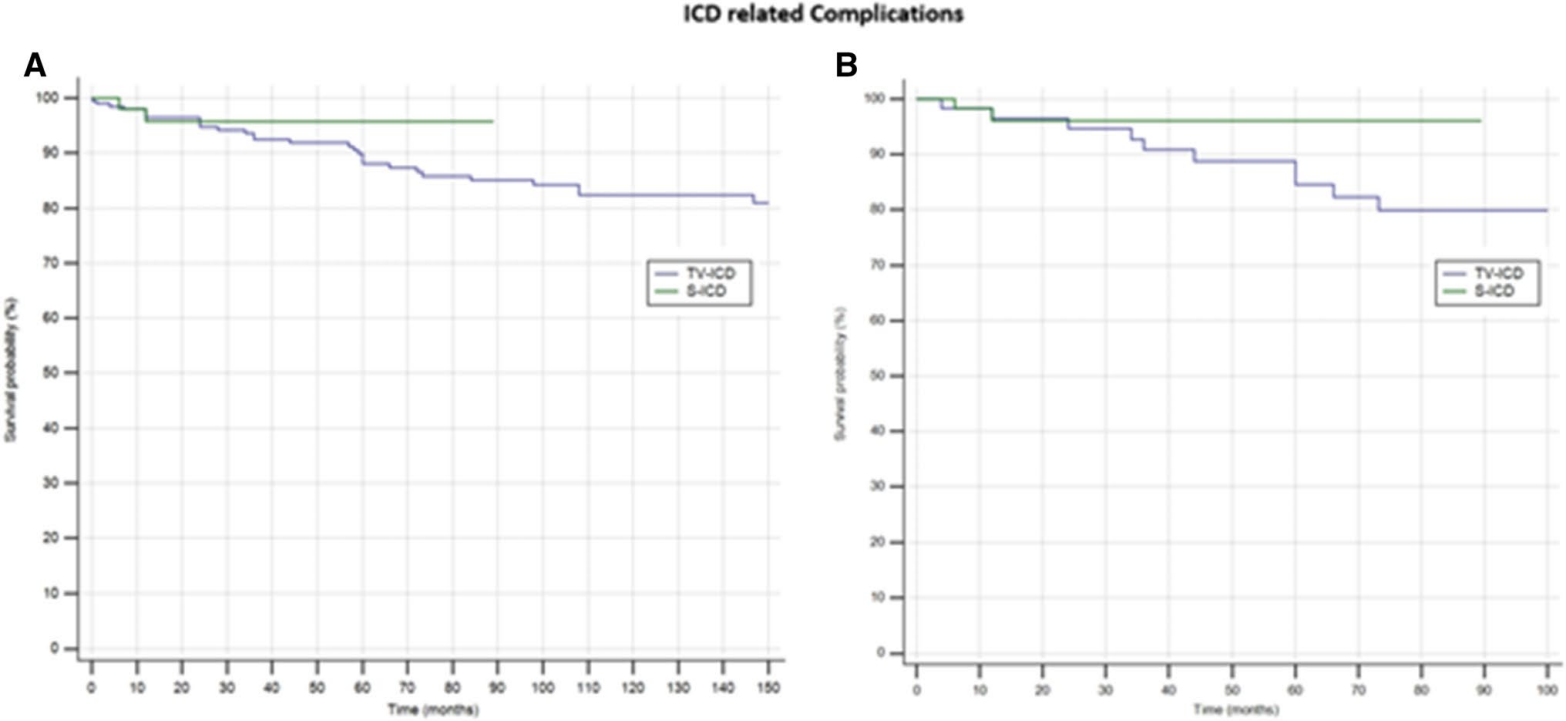
Kaplan–Meier curvecomparing survival from ICD-related complications among S-ICD vs TV-ICD groups in unmatched (**A**) and matched (**B**) cohorts

At Cox univariate analysis no baseline patients’ characteristic, including the S-ICD (OR: 0.74; 95% CI: 0.27–1.99; *P* = *0.55*), was associated with ICD-related complications. The Kaplan–Meier analysis did not show a significantly different risk of ICD complications between the two subgroups in both unmatched (*log-rank P* = *0.41*) and matched (*log-rank P* = *0.09*) cohorts (Fig. 2).

#### ICD-related infections

ICD-related infections in need of leads extraction occurred in 10 patients (3.88%); 9 (4.5%) in TV-ICD group and 1 (1.8%) in S-ICD group (*P* = *0.31*).The annual incident rate of ICD-related infections was 0.44%.At Cox univariate analysis, no baseline patients’ characteristic, including the S-ICD (OR: 0.35; 95% CI 0.037–3.22; *P* = *0.55*), was significantly associated with ICD-related infections.

**Fig. 3:**
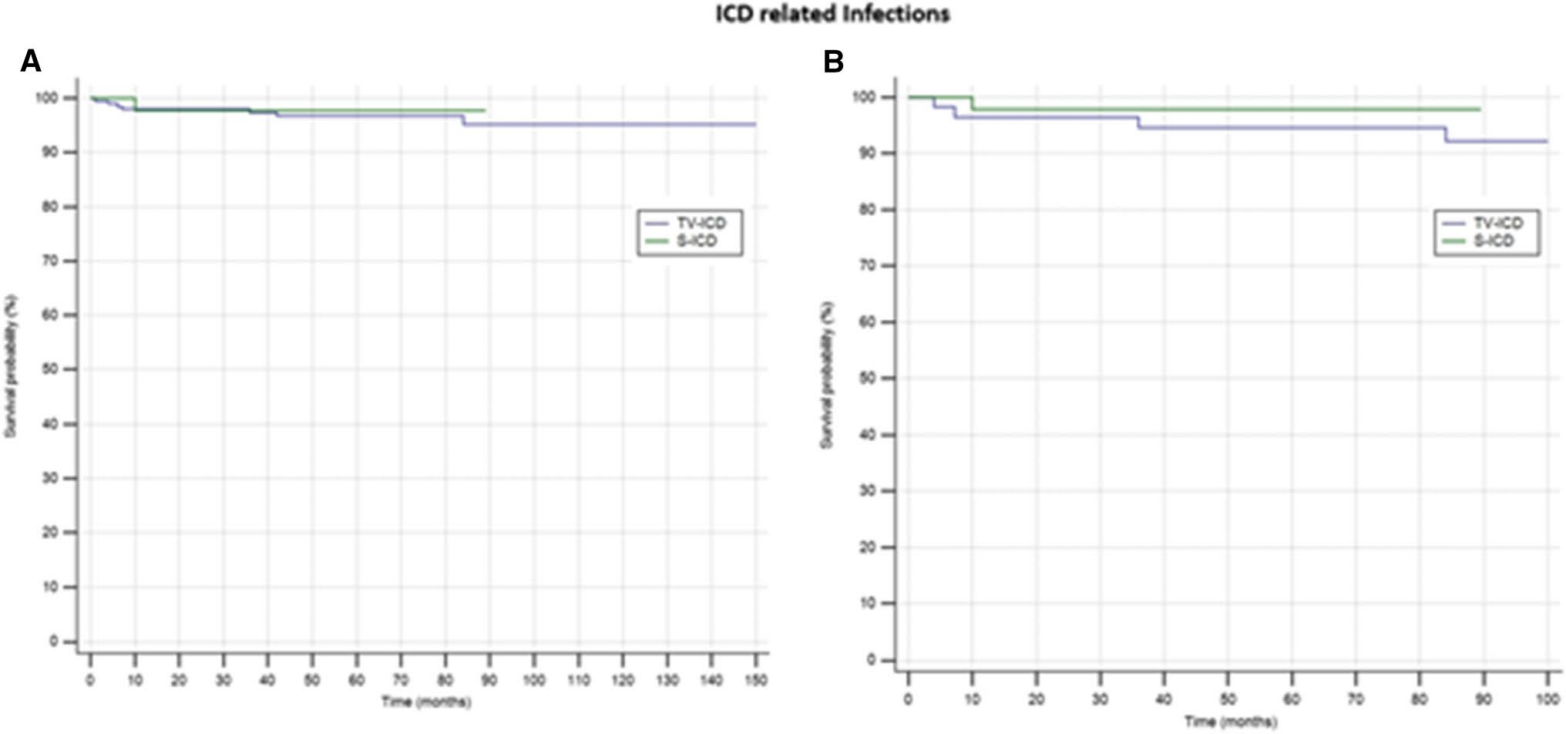
Kaplan–Meier curvecomparing survival from ICD-related infections among S-ICD vs TV-ICD groups in unmatched (**A**) and matched (**B**) cohorts

In the matched cohort, ICD-related infections occurred in 5 patients (4.16%); 4 (6.6%) in the TV-ICD group, and 1 (1.66%) in the S-ICD group (*P* = *0.166*). At Cox univariate analysis, the ICD replacement was the only independent predictor of ICD-related infectious (OR: 2.21; 95% CI 1.22- 4.01; *P* = *0.02*). The Kaplan–Meier analysis did not show a significantly different risk of ICD-related infections between the 2 subgroups in both matched (*log rank P* = *0.80*) and unmatched (*log rank P* = *0.33*) cohorts (Fig. 3).

#### All-cause mortality and appropriate ICD therapies

5 (1.9%) patients died during follow-up, all in the TV-ICD subgroup. The annual incident rate of mortality was 0.33%0.13 (5%) patients experienced appropriate ICD therapies, all in the TV-ICD subgroup. The annual incident rate of appropriate ICD therapies was 0.32%.

## Discussion

The main results of our study are the following: among drug-induced BrS patients no significant differences in inappropriate ICD therapies and ICD-related complications and infections were shown between TV-ICD vs S-ICD group; S-ICD was characterized by a lower rate of lead-related complications leading to surgical revision or extraction; no patients’ clinical features were associated to ICD inappropriate shocks or complications.The overall incidence of inappropriate ICD therapies among our cohort of drug-induced BrS patients was about 6.2% over a median follow-up of 83.7 months, three times lower than that emerged from a recent systematic review including 11 studies and 750 BrS patients, of whom 53.4% with drug-induced type-1 BrS, followed for 82.3 months [4]**.** Moreover, the annual incidence rate of inappropriate ICD therapies among our study population was 0.6%, 5 times lower than that emerged (3.3%) from a metanalysis of 22 studies including 1539 BrS patients with ICD, of whom 49% with drug-induced type-1 BrS [8].

The different incidence of ICD inappropriate therapies among our study population might be explained by different factors: first, our study cohort was composed of only drug-induced BrS patients. Considering that the presence of a spontaneous type 1 ECG pattern is one of the main causes of inappropriate shock among S-ICD patients [7], the exclusion of these patients from our study reduces this risk. We cannot exclude the probability of intermittent type 1 pattern, even if no specific triggers, such as fever or drug administration, were reported. Moreover, the introduction of an additional high-pass filter to the S-ICD sensing methodology [9], the enhanced supraventricular tachycardia discriminators [10], the activation of lead noise reduction algorithms [11], and the TV-ICD programming based on VF-only zone [12] with a cut-off rate greater than 220–240 bpm [13] and longer detection intervals [14] may have further reduced the risk of inappropriate ICD therapies.

The main causes of the inappropriate ICD therapies in our study cohort were the misdetection of supraventricular arrhythmias, mainly AF, in the TV-ICD group and the oversensing due to air entrapment in the S-ICD group. This latter evidence underlines the need to perform a systematic approach including device interrogation, provocative maneuvers, and chest radiography to early detect this early complication and an uncommon cause of IAS in S-ICD recipients [15].

Regarding the ICD-related complications, we observed an overall incidence of 12% over a median follow-up of 83.7 months, mainly lead-related, with an annual incident rate of 1.6%, as previously shown[8].

Among our study population, we showed lower ICD-related complications in S-ICD compared to TV-ICD recipients, mainly driven by less frequent lead-related complications; in contrast, no significant difference in device-related complications was shown between the 2 groups. These results confirm also in drug-induced type-1 BrS patients the emerging evidence from recent observational studies [16, 17].

Considering the young age of BrS patients, the reduction in lead-related complications represents a not negligible factor to consider in favor of the S-ICD implantation among this subgroup. Lead extraction is indeed one of the most hazardous electrophysiological procedures, accounting for risk of major complication rate of about 1.7%, including mortality of 0.5% [18]. The possibility to avoid transvenous lead implantation removes the risk of this type of complication.

Among our population, we reported a low rate of ICD infections, confirming the reduced number of infections in high implantation volume centers [19, 20]**.** This result may be due to the low prevalence of patients’risk factors for CIED infections, such as medical comorbidities, and the use of single-lead TV-ICD. Different from previous evidence [16, 17], no significant difference in ICD infections was shown between the TV-ICD and S-ICD groups. This evidence is of pivotal importance since systemic infections represent an important predictor of death for all causes, regardless of the result of the extraction procedure [21]. The only variable significantly associated with increased ICD-related infections in our population was the ICD replacement, as previously shown in the general population [22]. Moreover, it should be noted that the significant difference in the median follow-up duration among the two groups, 111 months in TV-ICD vs 46.3 months in S-ICD, might have underestimated the advantages of S-ICD. Looking at the Kaplan–Meier curves of all the outcomes, a clear separation between the two groups is evident, but a significant difference was not reached due to the low number of events and the shorter follow-up in the S-ICD group. This stresses the need of a longer follow-up which may help in better comprehending the long-term issue in this particular subgroup of Brugada patients.In light of all the above, our results support the hypothesis that S-ICD is at least not inferior to TV-ICD in drug-induced type-1 BrS patients; moreover, it offers an additive advantage in reducing the lead-related complications.

## Limitations

The retrospective nature and non-randomized comparison are certain a limitation. However, the present study was the first including exclusively patients with drug-induced BrS who underwent S-ICD and TV-ICD implantation. The significant difference in the follow-up time between the two groups and the low number of outcome events may havei nfluenced the significatively of our results. Indeed, a bias could derive from the differences between groups and specifically from factors influencing the operator's decision to implant S-ICD or TV-ICD. Finally, the large time frame in which TV-ICD patients were implanted could itself be a bias, as, there have been changes in device programming after 2007 to reduce the frequency of inappropriate therapies [23, 24].

## Conclusions

In a real-world setting of drug-induced type-1 BrS patients with ICD, no significant differences in inappropriate ICD therapies, device-related complications, and infections were shown among S-ICD vs TV-ICD. On the other hand, a reduction in lead-related complications was observed in the S-ICD group. Our evidence suggests that S-ICD is at least non-inferior to TV-ICD in this population and may also reduce the risk of lead-related complications which can expose the patients to the necessity of lead extractions.
