# Correction to: Subcutaneous versus transvenous implantable cardioverter-defibrillator among drug-induced type-1 ECG pattern Brugada syndrome: a propensity score matching analysis from IBRYD study

**DOI:** 10.1007/s00380-022-02228-3

**Published:** 2023-01-06

**Authors:** Vincenzo Russo, Alfredo Caturano, Federico Guerra, Federico Migliore, Giuseppe Mascia, Andrea Rossi, Martina Nesti, Vincenzo Ezio Santobuono, Emilio Attena, Gianfranco Tola, Luigi Sciarra, Giulio Conte, Alessandro Paoletti Perini, Pietro Francia, Gregory Dendramis, Zefferino Palamà, Stefano Albani, Andrea Ottonelli Ghidini, Leonardo Calò, Antonio D’Onofrio, Enrico Baldi, IBRYD Study Group, Gerardo Nigro, Ferdinando Carlo Sasso, Luca Barca, Italo Porto, Pasquale Notarstefano, Maria Antonietta Ruocco, Livia Franchetti Pardo, Carmen Adducci, Nicola Berlier, Berardo Sarubbi, Alessandro Vicentini, Roberto Floris, Emanuele Romeo, Paolo Golino, Nicolò Martini, Chiara Carrozzi

**Affiliations:** 1 Department of Medical Translational Sciences, Division of Cardiology, University of Campania “Luigi Vanvitelli”, Monaldi Hospital, Naples, Italy; 2 Department of Advanced Medical and Surgical Sciences, University of Campania “Luigi Vanvitelli”, Piazza Luigi Miraglia 2, T80138 Naples, Italy; 3 Azienda Ospedaliera Universitaria Ospedali Riuniti, Ancona, Italy; 4 Università degli Studi di Padova, Padua, Italy; 5 IRCCS San Martino Polyclinic Hospital, Genoa, Italy; 6 Gabriele Monasterio Foundation, Pisa, Italy; 7 Cardiovascular and Neurological Department, Ospedale San Donato, Via Nenni, 20/22, 52100 Arezzo, Italy; 8 Department of Interdisciplinary Medicine and Policlinico of Bari, Cardiology Unit, University of Bari “Aldo Moro”, Bari, Italy; 9 Cardiology Unit, Roccadaspide Hospital, ASL Salerno, Roccadaspide, Italy; 10 Azienda Ospedaliera Brotzu, Cagliari, Italy; 11 Policlinico Casilino, Cardiology Unit, Rome, Italy; 12 Cardiocentro Ticino Foundation, Lugano, Switzerland; 13 Azienda Sanitaria di Firenze, Florence, Italy; 14 Azienda Ospedaliero-Universitaria Sant’Andrea, Rome, Italy; 15 Clinical and Interventional Arrhythmology, Cardiology Unit, ARNAS, Ospedale Civico Di Cristina Benfratelli, Palermo, Italy; 16 Casa di Cura Villa Verde, Taranto, Italy; 17 Umberto Parini Regional Hospital, Aosta, Italy; 18 Versilia Hospital, Cardiology Unit, Lido di Camaiore (LU), Italy; 19 Departmental Unit of Electrophysiology, Evaluation and Treatment of Arrhythmias, Monaldi Hospital, Naples, Italy; 20 IRCCS Policlinico San Matteo, Pavia, Italy; 21 Clinical Cardiology, Department of Medical Sciences and Public Health, University of Cagliari, Cagliari, Italy; 22 Department of Cardiology, University of Campania “Luigi Vanvitelli”, Monaldi Hospital, Naples, Italy

**Correction to: Heart and Vessels** 10.1007/s00380-022-02204-x

In the original publication of the article, under acknowledgements, the below author’s names, affiliation information were missing and included in this article.

1. Nicolò Martini (Università degli Studi di Padova, Padua, Italy).

 2. Chiara Carrozzi (IRCCS Policlinico San Matteo, Pavia, Italy).

